# Derivation, validation, and transcriptomic assessment of pediatric septic shock phenotypes identified through latent profile analyses: Results from a prospective multi-center observational cohort

**DOI:** 10.21203/rs.3.rs-3692289/v1

**Published:** 2023-12-06

**Authors:** Mihir R. Atreya, Min Huang, Andrew R. Moore, Hong Zheng, Yehudit Hasin-Brumshtein, Julie C. Fitzgerald, Scott L. Weiss, Natalie Z. Cvijanovich, Michael T. Bigham, Parag N. Jain, Adam J. Schwarz, Riad Lutfi, Jeffrey Nowak, Neal J. Thomas, Michael Quasney, Mary K. Dahmer, Torrey Baines, Bereketeab Haileselassie, Andrew J. Lautz, Natalja L. Stanski, Stephen W. Standage, Jennifer M. Kaplan, Basilia Zingarelli, Timothy E. Sweeney, Purvesh Khatri, L. Nelson Sanchez-Pinto, Rishikesan Kamaleswaran

**Affiliations:** Division of Critical Care Medicine, Cincinnati Children’s Hospital Medical Center, Cincinnati, OH, 45229, USA.; Department of Pediatrics, University of Cincinnati College of Medicine, Cincinnati, OH, 45627, USA; Department of Biomedical Informatics, Emory University School of Medicine, Atlanta, GA, USA.; Stanford Institute for Immunity, Transplantation and Infection, Stanford University School of Medicine, Stanford, CA; Stanford Institute for Immunity, Transplantation and Infection, Stanford University School of Medicine, Stanford, CA.; Center for Biomedical Informatics Research, Department of Medicine, Stanford University School of Medicine, Stanford, 94305, CA.; Inflammatix, Sunnyvale, CA, 94085, USA.; Children’s Hospital of Philadelphia, Philadelphia, PA, 19104, USA.; Nemours Children’s Health, Wilmington, DE, 19803, USA; UCSF Benioff Children’s Hospital Oakland, Oakland, CA, 94609, USA.; Akron Children’s Hospital, Akron, OH, 44308, USA.; Texas Children’s Hospital, Baylor College of Medicine, Houston, TX, 77030, USA.; Children’s Hospital of Orange County, Orange, CA, 92868, USA.; Riley Hospital for Children, Indianapolis, IN, 46202, USA.; Children’s Hospital and Clinics of Minnesota, Minneapolis, MN, 55404, USA.; Penn State Hershey Children’s Hospital, Hershey, PA, 17033, USA.; C.S Mott Children’s Hospital, University of Michigan, Ann Arbor, MI, 48109, USA.; C.S Mott Children’s Hospital, University of Michigan, Ann Arbor, MI, 48109, USA.; University of Florida Health Shands Children’s Hospital, Gainesville, FL, 32610, USA.; Lucile Packard Children’s Hospital Stanford, Palo Alto, CA, 94304, USA.; Division of Critical Care Medicine, Cincinnati Children’s Hospital Medical Center, Cincinnati, OH, 45229, USA.; Department of Pediatrics, University of Cincinnati College of Medicine, Cincinnati, OH, 45627, USA; Division of Critical Care Medicine, Cincinnati Children’s Hospital Medical Center, Cincinnati, OH, 45229, USA.; Department of Pediatrics, University of Cincinnati College of Medicine, Cincinnati, OH, 45627, USA; Division of Critical Care Medicine, Cincinnati Children’s Hospital Medical Center, Cincinnati, OH, 45229, USA.; Department of Pediatrics, University of Cincinnati College of Medicine, Cincinnati, OH, 45627, USA; Division of Critical Care Medicine, Cincinnati Children’s Hospital Medical Center, Cincinnati, OH, 45229, USA.; Department of Pediatrics, University of Cincinnati College of Medicine, Cincinnati, OH, 45627, USA; Division of Critical Care Medicine, Cincinnati Children’s Hospital Medical Center, Cincinnati, OH, 45229, USA.; Department of Pediatrics, University of Cincinnati College of Medicine, Cincinnati, OH, 45627, USA; Inflammatix, Sunnyvale, CA, 94085, USA; Stanford Institute for Immunity, Transplantation and Infection, Stanford University School of Medicine, Stanford, CA.; Center for Biomedical Informatics Research, Department of Medicine, Stanford University School of Medicine, Stanford, 94305, CA.; Department of Pediatrics, Northwestern University Feinberg School of Medicine, Chicago, 60611, IL, USA.; Department of Health and Biomedical Informatics, Northwestern University Feinberg School of Medicine, Chicago, 60611, IL, USA.; Department of Biomedical Informatics, Emory University School of Medicine, Atlanta, 30322, GA, USA.; Department of Biomedical Engineering, Georgia Institute of Technology, Atlanta, 30322, GA, USA.

**Keywords:** Sepsis, Septic shock, Precision Medicine, Multiple Organ Dysfunction, Latent Profile Analyses, Biomarkers, Innate immunity, Adaptive immunity, Endothelial dysfunction, Gene-expression, Endotype, Phenotype, Endophenotype

## Abstract

**Background:**

Sepsis poses a grave threat, especially among children, but treatments are limited due to clinical and biological heterogeneity among patients. Thus, there is an urgent need for precise subclassification of patients to guide therapeutic interventions.

**Methods:**

We used clinical, laboratory, and biomarker data from a prospective multi-center pediatric septic shock cohort to derive phenotypes using latent profile analyses. Thereafter, we trained a support vector machine model to assign phenotypes in a hold-out validation set. We tested interactions between phenotypes and common sepsis therapies on clinical outcomes and conducted transcriptomic analyses to better understand the phenotype-specific biology. Finally, we compared whether newly identified phenotypes overlapped with established gene-expression endotypes and tested the utility of an integrated subclassification scheme.

**Findings:**

Among 1,071 patients included, we identified two phenotypes which we named ‘inflamed’ (19.5%) and an ‘uninflamed’ phenotype (80.5%). The ‘inflamed’ phenotype had an over 4-fold risk of 28-day mortality relative to those ‘uninflamed’. Transcriptomic analysis revealed overexpression of genes implicated in the innate immune response and suggested an overabundance of developing neutrophils, pro-T/NK cells, and NK cells among those ‘inflamed’. There was no significant overlap between endotypes and phenotypes. However, an integrated subclassification scheme demonstrated varying survival probabilities when comparing endophenotypes.

**Interpretation:**

Our research underscores the reproducibility of latent profile analyses to identify clinical and biologically informative pediatric septic shock phenotypes with high prognostic relevance. Pending validation, an integrated subclassification scheme, reflective of the different facets of the host response, holds promise to inform targeted intervention among those critically ill.

## Introduction

Sepsis is defined as life-threatening organ dysfunction caused by a dysregulated host response to an infection. It represents a major public health problem, especially among children, where it affects an estimated 20 million each year across the globe. (1) Moreover, sepsis is the leading cause of under-five mortality. (2) Yet, despite numerous clinical trials, sepsis care remains limited to early antibiotics and intensive organ support. This lack of therapeutic efficacy has been attributed, in part, to the heterogeneity among critically ill patients. (3) Thus, reproducible approaches that identify clinically and biologically relevant subclasses are necessary to facilitate targeted therapeutic approaches and ultimately to improve patient outcomes. (4)

Gene-expression profiling of whole blood has been used to identify sepsis subclasses. (5–8) Among children, *Wong* and colleagues used a 100 gene-expression panel, to identify pediatric septic shock endotypes – A and B with prognostic value; assignment to endotype A was associated with a nearly 3-fold increased risk of mortality, relative to those with endotype B. (9) Subsequently, these endotypes were shown to demonstrate a differential response to corticosteroids in observational studies, with patients classified as endotype A having a 4-fold increase in mortality with corticosteroid use, relative to patients with endotype B. (10) Similar strategies have been deployed among adults yielding analogous results. (11) Of note, gene-expression-based endotyping is being tested in the ongoing *Stress Hydrocortisone in Pediatric Septic Shock* (SHIPPS, NCT03401398) trial and holds promise to demonstrate the feasibility of employing predictive enrichment strategies among critically ill children.

Concomitantly, a decade ago, *Calfee* et al. leveraged latent class analyses of clinical, laboratory, and biomarker data to identify two phenotypes of acute respiratory distress syndrome (ARDS). The *hyperinflammatory* group was characterized by worse outcomes, relative to those without this phenotype. (12) Of note, these phenotypes have demonstrated heterogeneity in treatment effect (HTE) in response to several interventions in secondary analyses of ARDS trials (12, 13), and corticosteroids among critically ill COVID-19 patients. (14) More recently, *Dahmer* et al. and others have shown reproducibility and prognostic utility of this approach among children with ARDS. (15, 16) Lastly, using similar approaches, *Sinha* and colleagues recently published on molecular phenotypes among adults with sepsis. (17) To the best of our knowledge, no study has evaluated the reproducibility of latent profile phenotypes in pediatric sepsis.

In the current study, we sought to derive pediatric septic shock phenotypes using latent profile analyses and test their reproducibility in our longstanding multi-center prospective observational cohort based in the U.S. We sought to establish their prognostic value and to test for interactions between phenotypic and commonly used interventions against sepsis on clinically relevant outcomes. To establish their biological significance, we conducted transcriptomic analyses in a subset of the cohort to identify differentially expressed genes and infer cell populations linked to phenotypes. Lastly, we compared the overlap between established gene-expression endotypes of pediatric septic shock and newly identified latent profile phenotypes. We tested the hypothesis that integrating endotype and phenotype assignments could provide a refined framework for the subclassification of critically ill children.

## Methods

### Study design and patient selection

Our ongoing prospective observational cohort study of pediatric septic shock has been extensively detailed previously. (10, 18, 19) Inclusion criteria for study enrollment was all patients meeting consensus criteria for pediatric septic shock (20) recruited between 2003 and 2023 from 13 pediatric intensive care units (PICUs) in the U.S. Blood was collected from consenting participants within 24 hours of meeting enrollment criteria (day 1). Patients who did not require any vasoactive support were excluded. The primary outcomes of interest included 7- and 28- day mortality, and complicated course – a composite endpoint of death by or presence of ≥ 2 organ dysfunctions on day 7 after study enrollment.

### Derivation set

We randomly split patients in the cohort into derivation (60%) and hold-out validation (40%) sets. We used R package “mclust” (v.6.0.0) to perform latent profile analyses – a Gaussian Finite Mixture Modeling approach– using clinical, laboratory, and biomarker variables in the derivation set. Briefly, we included deviation of vital signs from the median values for age and sex during health. Laboratory data were obtained at the discretion of treating physicians. Biomarker data were previously measured using multiplex Luminex assays in serum collected on day 1. Additional details and selection of the number of latent profiles are detailed in the **Online Supplement.**

### Validation set

The phenotype assignments in the derivation set were used to train a support vector machine (SVM) classifier, which was used to assign phenotypes in the validation set. We compared patient demographics, characteristics, outcomes, and biomarkers, in the derivation and validation sets to test reproducibility and ensure clinical and biological relevance of assigned phenotypes.

### Transcriptomic analyses

Bulk messenger RNA sequencing data was available from a subset of the cohort recruited between 2019 and 2023 from day 1 biospecimens. We used DESeq2 (v.1.38.3) to identify differentially expressed genes (DEGs) between the latent profile phenotypes. DEGs were selected based on ≥ log2 fold change value cutoff of ± 1, and adjusted p-value of 0.05. We conducted Reactome pathway analyses with a Benjamin Hochberg false discovery rate (FDR) < 0.05 to identify enriched biological pathways and CIBERSORT analyses, a bulk deconvolution approach, to determine differences in cell subsets between phenotypes.

### Inference of cell types underlying phenotypes

We sought to gain granular insight at a single-cell level into immune cell subpopulations associated with latent profile phenotypes. To achieve this, we first integrated three single-cell RNA sequencing datasets, which included data on neutrophil subsets among critically ill adults, a vast majority of whom had COVID-19. (21–23) We calculated a composite gene score by subtracting the geometric mean of underexpressed genes from the geometric mean of overexpressed genes, identified through DEG analyses comparing phenotypes. We inferred differences in the abundance of cell subsets between phenotypes by referencing the composite gene score against the integrated single-cell dataset.

### Comparison with established gene-expression pediatric septic shock endotypes

A subset of patients in the cohort had existing assignments as endotypes A or B based on historical data using a 100-gene panel on the Nanostring nCounter platform. Briefly, image analysis of gene-expression mosaics were previously used to assign pediatric septic shock endotypes, with endotype A being characterized by a repressed adaptive immune response relative to endotype B. (10)

#### Statistical analyses:

We assessed differences in demographic and clinical characteristics between groups by non-parametric Kruskal-Wallis tests for continuous variables and χ2 tests for categorical variables. Multivariate logistic regression models were used to assess the association between phenotype and outcomes of interest and adjusted for era of enrollment (2013–2023 vs. 2003–2012), patient age, pediatric risk of mortality score (PRISM III), (24) presence of comorbidity, and immunocompromised status. Interactions between phenotype and commonly used sepsis therapies on clinical outcomes were tested based on results of binary logistic regression models adjusted for age and PRISM III score. Pearson χ2 test was used to test the overlap between established gene-expression endotypes and latent profile phenotypes. Kaplan Meier curves were used to estimate differences in survival comparing endotypes, phenotypes, and an integrated subclass assignment scheme where we considered outputs of both these approaches. The relative risk of 28-day mortality among subclasses was compared by Cox regression analyses. A two-tailed p-value < 0.05 was used to test significance. **Role of the funding source:** The content is solely the responsibility of the authors and does not necessarily represent the official views of the NIH (U.S.).

## Results

The overview of the study and analyses is detailed in **Supplementary Fig. 1**. A total of 1,395 patients met the inclusion criteria for the study of whom we excluded 324 patients who did not receive any vasoactive support. The median age of the patients included in the study (n = 1,071) was 5.3 years (quartile 1: 1.7; quartile 3: 11.0 years). The derivation set was comprised of 646 patients and the validation set included 425 patients.

**Figure 1:**
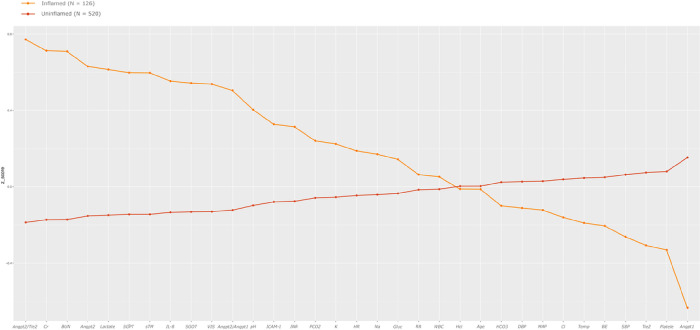
Standardized mean (z-scores) for continuous class predicting variables in the derivation set by latent profile is shown on the y-axis. The predictor variables are sorted on the x-axis from left to right in descending order of difference between the ‘inflamed’ (shown in orange) and ‘uninflamed’ (shown in brown) phenotypes. Angpt2/Tie-2: Angiopoietin-2/Tie-2 ratio; Cr: Creatinine; BUN: blood urea nitrogen; Angpt-2: Angiopoietin-2; Lactate: Serum lactate; SGPT: serum glutamic pyruvic transaminase; sTM: soluble Thrombomodulin; IL-8: Interleukin-8; SGOT: serum glutamic-oxaloacetic transaminase; VIS: Max vasoactive inotropic score on day 1; Angpt-2/Angpt-1: Angiopoietin-2/Angiopoietin-1 ratio; pH; ICAM-1: Intercellular adhesion molecule 1; INR: international normalized ratio; PCO2: partial pressure of carbon dioxide; K: potassium; HR: deviation from age and sex normalized heart rate; Na: Sodium; Gluc: Glucose; RR: respiratory rate; WBC: white blood cell count; HCt: hematocrit; Age: age in years; HCO3: serum bicarbonate; DBP: diastolic blood pressure; MAP: mean arterial pressure; Cl: serum chloride; Temp: Temperature; BE: base excess; SBP: systolic blood pressure; Tie-2: tyrosine kinase with immunoglobulin-like loops and epidermal growth factor homology domains-2; Platelet: platelet count; Angpt-1: Angiopoietin-1.

Latent profile analyses in the derivation set revealed two phenotypes. Differences in standardized variables between the two phenotypes are shown in Fig. 1. One of the phenotypes (n = 126, 19.5%) was characterized by high Angiopoietin-2/Tie-2 ratio, Angiopoietint-2, soluble thrombomodulin (sTM), interleukin 8 (IL-8), and intercellular adhesion molecule 1 (ICAM-1) and low Tie-2 and Angiopoietin-1, which we designated as the ‘inflamed’ phenotype. This group was characterized by a high serum creatinine, blood urea nitrogen (BUN), lactate, a high international normalized ratio (INR), and low platelet counts. We labeled the remaining patients (n = 520, 80.5%), characterized by the absence of such features, as the ‘uninflamed’ phenotype.

Table 1 shows the comparisons between phenotypes in the derivation and validation sets – the latter based on the assignments of our SVM classifier. There were no differences in age and sex comparing phenotypes. Although patients who were ‘inflamed’ were more likely to have had a history of oncologic disease or bone marrow transplantation than those ‘uninflamed’ in the derivation set, there were no statistically significant differences in the validation set. Patients with an ‘inflamed’ phenotype had a trend toward higher rates of positive blood cultures in the derivation set (26.2% vs. 19.2%, p = 0.08), which reached statistical significance in the validation set (33.8% vs. 20.6%, p = 0.016), relative to those ‘uninflamed’. There were no significant differences in the type of pathogen. Patients with an ‘inflamed’ phenotype had higher baseline illness severity and significantly worse clinical outcomes in the derivation and validation sets. Finally, patients with an ‘inflamed’ phenotype were more likely to have been prescribed adjunctive corticosteroids by treating physicians, relative to those ‘uninflamed’.

Patients with an ‘inflamed’ phenotype had over 5-fold higher odds of 7-day mortality (adj. OR 5.6, 95% CI: 3.6–8.6, p < 0.001), over 4-fold higher odds of 28-day mortality (adj. OR 4.4, 95% CI: 3.0–6.4, p < 0.001), and nearly 4-fold higher odds of complicated course (adj. OR 3.9, 95% CI: 2.8–5.5, p < 0.001) relative to those ‘uninflamed’. Results of interactions between phenotypes and common sepsis therapies on patient outcomes are detailed in Table 2. Patients with an ‘inflamed’ phenotype were more likely to have received ≥ 100 ml/kg of fluid on day 1 of PICU admission, ≥ 2 vasoactive agents, corticosteroids, required intubation and continuous renal replacement therapy (CRRT) support with commensurately worse outcomes, relative to those who ‘uninflamed’. We did not identify any significant interaction between phenotype and sepsis therapies on outcomes with one exception. Patients with an ‘inflamed’ phenotype who received ≥ 2 antimicrobial therapies had a significantly higher rate of complicated course in comparison with those ‘uninflamed’ who received ≥ 2 antimicrobial therapies (65.5% vs 26.6%, interaction p-value 0.021).

**Figure 2:**
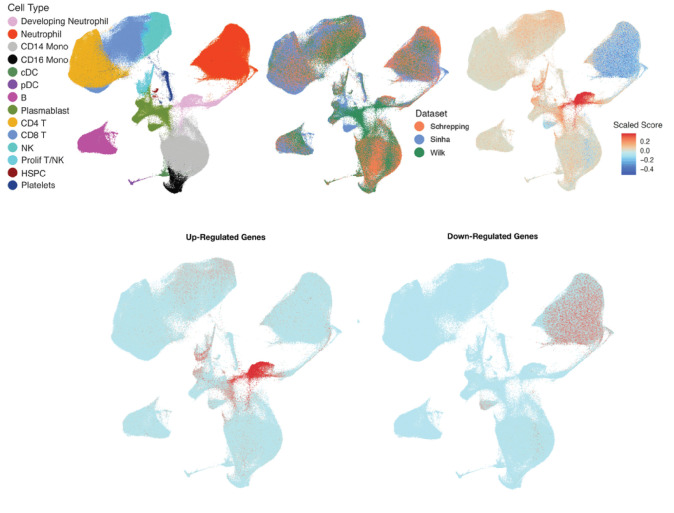
Inference of cell subsets underlying pediatric septic shock phenotypes identified in the study. The figures show the Uniform Manifold Approximation and Projection (UMAP) of the integrated single-cell transcriptomic dataset from critically ill patients including adults with COVID-19. Top panel from left to right. (a.) Fourteen cell subsets were identified in the integrated dataset. (1) Developing neutrophils (pink), (2) Mature neutrophils (red), (3) Cluster differentiation (CD) 14 positive monocytes (light gray), (4) CD16 positive monocytes (black), (5) cDC: classic dendritic cells (green), (6) pDC: plasmacytoid dendritic cells (purple), (7) B lymphocytes (magenta), (8) PB: Plasmablasts (moss green), (9) CD4 positive T lymphocytes (yellow), (10) CD8 positive T lymphocytes (sky blue), (11) NK: Natural killer cells (light teal), (12) Pro T/NK: Pro T lymphocytes/natural killer cells (aqua), (13) HSPC: hematopoietic stem and progenitor cells (maroon), and (14) Platelets (dark blue). (b.) Origin of cells in the integrated single-cell map based on the dataset (1) Schrepping et al, Cell, 2020 (orange), (2) Sinha et al. Nature Medicine, 2022 (blue), (3) Wilk, J. Exp. Medicine 2021 (green). (c.) Composite gene score calculated by subtracting the geometric mean of underexpressed genes (*PRLR, HCAR2, RAMP3, SHE, and CMTM2*) from the geometric mean of overexpressed genes (*CCL4, PRTN3, NEIL3, CENPU, ELANE, SKA3, CEP55, NCAPH, HBB, DEFA4, DEFA3, CTSG, CCL20*, and *MMP15*) among patients with an ‘inflamed’ phenotype relative to those ‘uninflamed’ projected on the UMAP of the integrated single-cell dataset. The gene score was scaled as shown in the legend with genes in red representing those overexpressed and those in blue showing those underexpressed. Bottom panel from left to right. (d.) Projection of overexpressed genes identified among patients with an ‘inflamed’ phenotype relative to those ‘uninflamed’ on the integrated single-cell dataset demonstrated that genes corresponded to signatures of developing neutrophils, proliferating T/NK cells, and NK cells. (e.) Projection of underexpressed genes identified among patients with an ‘inflamed’ phenotype relative to those ‘uninflamed’ on the integrated single-cell dataset demonstrated that genes corresponded to signatures of mature neutrophils.

Transcriptomic data was available in 144 patients. We identified 44 differentially expressed genes (DEGs) when comparing patients with ‘inflamed’ (n = 17) vs. ‘uninflamed’ phenotype (n = 127), of which 25 genes were overexpressed and 19 were underexpressed. Biological pathways enriched among patients with an ‘inflamed’ phenotype relative to those ‘uninflamed’ corresponded to activation of the immune system, cytokine signaling, neutrophil degranulation, and antimicrobial peptides. CIBERSORT analyses identified that the proportion of neutrophils was lower among patients with an ‘inflamed’ phenotype relative to those ‘uninflamed’. Expression data was available for 14 overexpressed and 5 underexpressed genes, identified through DEG analyses, in the integrated single-cell dataset. After correction for multiple comparisons, genes overexpressed among those with an ‘inflamed’ phenotype corresponded to those expressed by developing neutrophils, proliferating T lymphocytes/Natural Killer (NK) cells, and NK cells. In contrast, genes underexpressed among those with an ‘inflamed’ phenotype corresponded to those expressed by mature neutrophils. These data are shown in Fig. 2; with additional details presented in the **Online Supplement**.

**Figure 3:**
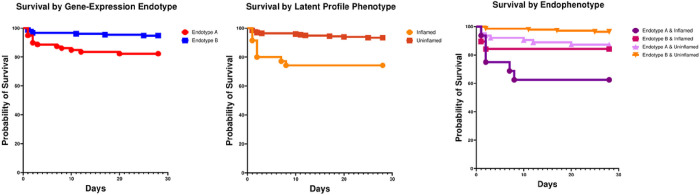
Kaplan Meier survival curves from left to right based on (a) established gene-expression endotype (A in red vs. B in blue), (2) latent profile phenotype (‘inflamed in orange and ‘uninflamed’ in brown), and (3) an integrated subclass assignment scheme that considered both the endotype and phenotype assignment among individual patients. The latter included all four possible combinations including (i) endotype A/inflamed (deep purple), (ii) endotype B/inflamed (deep plum), (iii) endotype A/uninflamed (light magenta), (iv) endotype B/uninflamed (orange). Patients with endotype A had a higher relative risk of 28-day mortality compared to endotype B (RR 3.7 (95% CI: 1.5, 8.7), p=0.003). Patients with an ‘inflamed’ phenotype had a higher relative risk of 28-day mortality compared to those with an ‘uninflamed’ phenotype (RR 4.5 (95% CI: 1.9, 10.6), p<0.001). Patients assigned as both endotype B and ‘uninflamed’ had the lowest mortality risk. Compared to this group, patients classified as endotype A & inflamed had a higher relative risk of mortality (RR 12.5 (95%CI: 3.8, 41.2), p <0.001). Patients classified as endotype B & ‘inflamed’ had a relative risk of mortality of 4.8 (95% CI: 1.1, 20.1, p=0.032). Patients classified as endotype A & uninflamed had a relative risk of mortality of 3.6 (95%CI: 1.2, 11.1), p=0.024. There were no statistically significant differences between the latter two groups.

A total of 233 patients in the study had data on established gene-expression endotype and latent profile phenotype assignments. There was no statistically significant association between endotypes and phenotypes in the cohort (Pearson χ2 test, p-value of 0.08). Figure 3 shows the Kaplan Meier survival curves based on gene-expression endotype (A vs. B), latent profile phenotype (‘inflamed’ vs. ‘uninflamed’), and an integrated scheme where we considered all four possible combinations of endotype and phenotype assignment. Patients classified as endotype B & ‘uninflamed’ had the lowest mortality risk. Relative to this group, those classified as endotype A & ‘inflamed’ had an over 12-fold (RR: 12.5, 95% CI: 3.8, 41.2, p < 0.001) higher relative risk of mortality; those with endotype B & ‘inflamed’ had a nearly 5-fold increase in mortality (RR; 4.8, 95% CI: 1.1, 20.1, p = 0.032); those with endotype A & ‘uninflamed’ had an over 3-fold increase in mortality (RR: 3.6, 95%CI: 1.2, 11.1, p = 0.024). There were no statistically significant differences in mortality between the latter two subclasses.

## Discussion

In this study, we derived and internally validated two pediatric septic shock phenotypes, identified through latent profile analyses, of high prognostic relevance. With one exception, there was no evidence for heterogeneous responses to common sepsis treatments on clinical outcomes between phenotypes. Transcriptomic analyses revealed overexpression of genes implicated in innate immune response among those with an ‘inflamed’ phenotype. Our data suggest a high turnover of neutrophils among this high-risk subset of patients, with additional roles for proliferating T/NK, and NK cells. We did not identify a significant overlap between established gene-expression endotypes and the newly derived latent profile phenotypes. Finally, we demonstrated the prognostic relevance of patient ‘endophenotypes’ based on an integrated subclassification scheme that considered both gene-expression-based endotypes and latent profile phenotypes.

The phenotypes identified in our study share similarities with the *hyper*- and *hypo-inflammatory* phenotypes originally described by Calfee and colleagues among adults with ARDS, (12, 13) and subsequently reproduced among pediatric patients; (15) the *reactive* and *uninflamed* phenotypes detailed by *Heijnen* et al. among mechanically ventilated adults; (25) molecular phenotypes of acute kidney injury detailed by *Bhatraju* et al. among adults; (26) and most recently those identified by Sinha et al. among septic adults. (17) Our data provide further support of the reproducibility of latent profile analyses as a methodologic approach to identify phenotypes, irrespective of assigned ‘syndromic’ diagnoses, across the spectrum of the host developmental age.

We provide evidence for the prognostic utility of latent profile phenotypes with the ‘inflamed’ group being independently associated with significant risk of poor clinical outcomes upon adjusting for multiple potential confounders. Unlike previous studies, beyond the robust prognostic implications, we did not find evidence of HTE of common sepsis therapies on clinical outcomes among phenotypes. The exception to this was that those patients with an ‘inflamed’ phenotype who received ≥ 2 antimicrobial therapies had significantly higher rate of complicated course than those with an ‘uninflamed’ phenotype. While this observation may merely reflect the fact that the ‘inflamed’ phenotype represented the sickest subset of patients, a few additional considerations are warranted (a) a lack of appropriate source control, (b) an inability to achieve therapeutic drug levels of antimicrobials and/or (c) an exaggerated host immune response, despite appropriate antimicrobial coverage, among those ‘inflamed’. Of note, our findings mirror those of *Sinha* et al. where the authors identified that septic adults with a *hyperinflammatory* phenotype had higher rates of bacteremia than those without. (17) Pending validation, future studies are needed to determine whether precision antibiotic dosing, targeted use of extra-corporeal blood purification strategies, and or modulation of the innate immune response can improve outcomes among patients with an ‘inflamed’ phenotype.

We did not identify a differential response to corticosteroids among phenotypes unlike that observed among adults with COVID-19. (14) The explanations for this difference are likely multifactorial including the relative homogeneity among patients with COVID-19 compared to the cohort studied, differences in pathogen type -viral vs. bacterial induced host response, and compartmentalized effects of corticosteroids based on primary cells affected - lung vs. peripheral blood. In addition, *Sinha* and colleagues demonstrate differential responses to recombinant activated protein C (rAPC) vs. placebo among phenotypes when re-examining results of the PROWESS-SHOCK trial data. (17) While we demonstrate evidence of a coagulopathy among those with an ‘inflamed’ phenotype, we cannot comment on whether latent profile phenotypes among children would be expected to have a similar biological response as with adults, given the developmental differences in host response.

Transcriptomic analyses revealed activation of neutrophil pathways consistent with gene-expression studies comparing phenotypes of adult ARDS and patients with sepsis as detailed by *Bos* et al. (27) Our data suggest a higher turnover of neutrophils among those with an ‘inflamed’ phenotype, as indicated by the signatures reflective of developing neutrophils relative to those ‘uninflamed’. A recent prospective single-cell multi-omics study by *Kwok* et al. among septic adults corroborates our data, wherein patients with the worst clinical outcomes were characterized by emergency granulopoiesis and the presence of immature neutrophils. (28) Finally, our data suggest a preponderance of additional cell subsets including proliferating T/NK and NK cells among those with an ‘inflamed’ phenotype. While we cannot confidently speak to whether the phenotypes identified represent ‘treatable traits’, (29) our data indicate that the groups identified are biologically distinct. Future studies are necessary to determine the mechanistic link between cell subpopulations and phenotypes, and whether targeted modulation of cell subsets can be used as a novel therapeutic approach against sepsis.

We did not identify a significant overlap between established gene-expression-based endotypes and latent profile phenotypes. As such our data indicate that, fundamentally, these two approaches are sampling different, albeit vitally important, biological facets of the host response in critical illness. While the former broadly reflects the adaptive arm of the host immune response, the latter informs the innate arm of the host response, including microvascular endothelial function. Therefore, we believe that the integrated classification scheme of ‘endophenotypes’ detailed in our study is of clinical and potential therapeutic relevance. For instance, patients classified as endotype A & ‘inflamed’ may represent an extreme endophenotype with a significantly increased risk of mortality. This is consistent with the observation that critically ill patients with an overactive innate- and repressed adaptive-immune response have been consistently associated with the worst clinical outcomes. As such these patients would be expected to be poor candidates to receive corticosteroids based on their endotype. However, they may potentially benefit from targeted immunomodulation to quell the innate immune response based on their phenotypic assignment. Furthermore, although patients with endotype B & ‘inflamed’ and endotype A & ‘uninflamed’ endophenotypes had comparably elevated risk of mortality, the therapeutic implication of such subclass assignment is expected to be diametrically opposite between groups. Although speculative, pending validation in cohort studies and clinical trials, such an integrated subclassification scheme holds the potential to inform better alignment of interventions among those critically ill by providing a comprehensive understanding of patient pathobiology. (30)

Our study has several limitations: (1) the observational nature of the study limits precludes any inference of causality; (2) despite accounting for era of patient enrollment in our multivariate models, the long study period is a limitation; (3) latent profile phenotypes only considered day 1 data. However, given the temporal and dynamic nature of the host response, it is conceivable that these class assignments may be subject to change over time; (4) external validation dataset to demonstrate the reproducibility of our SVM model was lacking. Moreover, we did not seek to develop a classifier that used a parsimonious set of predictor variables as this is better achieved in external validation sets; (5) the number of patients with an ‘inflamed’ phenotype among whom transcriptomic data was available was limited, which may have contributed to fewer DEGs being identified; (6) the integrated single-cell data used as reference was largely comprised of samples obtained from adults with COVID19 critical illness. Given that few single-cell studies to date have captured neutrophil signatures among septic patients, prospective studies that simultaneously capture phenotypic and single-cell transcriptomic data are necessary to directly identify cell subsets underlying phenotypes; (7) the number of patients in whom both established gene-expression endotype and latent profile phenotype class assignments were available was limited; 8) both endotype and phenotype assignments were based on data generated within 24 hours of meeting septic shock criteria and were assumed to reflect baseline differences in host response. However, a significant proportion of patients in the cohort received corticosteroids. It remains plausible that the biological differences in host response among subclasses may reflect those in response to corticosteroids, rather than baseline differences.

## Conclusions

In this study, we demonstrate the existence of two phenotypes among children with septic shock identified through latent profile analyses with high prognostic value. We provide evidence of upregulated innate immune responses among those with an inflamed phenotype reflective of signatures of developing neutrophils, proliferating T/NK, and NK cells. The phenotypes did not show overlap with established gene-expression-based adaptive endotypes in pediatric septic shock nor demonstrate a differential response to corticosteroids. We integrated these two promising classification schemes to delineate novel sepsis ‘endophenotypes’. Pending validation, such an approach may allow for therapeutic drug selection informed by a comprehensive understanding of patient-level pathobiology.

## Figures and Tables

**Table 1.** Demographics, patient characteristics, and clinical outcomes among pediatric septic shock latent profile phenotypes in the derivation and validation sets.

	Derivation set (n=646)		P value	Validation set (n=425)		P value
	Inflamed (n=126)	Uninflamed (n=520)		Inflamed (n=71)	Uninflamed (n=354)	
Age (Years)	4.7 (1.3, 13.7)	5.4 (1.8, 10.8)	0.698	6.2 (1.8, 14.0)	5.5 (1.8, 10.4)	0.480
Sex (Female)	57 (45.2%)	246 (47.3%)	0.676	39 (54.9%)	174 (49.2%)	0.374
Race			0.924			0.439
White or Caucasian	89 (70.7%)	376 (72.3%)		55 (77.4%)	263 (74.3%)	
Black or African American	16 (12.7%)	64 (12.3%)		6 (8.4%)	49 (13.8%)	
Other	21 (16.7%)	80 (15.4%)		10 (14.1%)	42 (11.9%)	
Ethnicity			0.214			0.063
Hispanic or Latino	12 (9.5%)	71 (13.6%)		3 (4.2%)	41 (11.6%)	
Non-Hispanic	114 (90.5%)	449 (86.4%)		68 (95.7%)	313 (88.4%)	
Culture:						
Any positive culture	71 (56.4%)	309 (59.4%)	0.529	44 (61.9%)	198 (55.9%)	0.348
Pulmonary	23 (18.2%)	133 (25.6%)		13 (18.3%)	68 (19.2%)	
Extra-pulmonary	48 (38.1%)	175 (33.6%)		31 (43.7%)	130 36.7%)	
Positive blood culture	33 (26.2%)	100 (19.2%)	0.083	24 (33.8%)	73 (20.6%)	0.016
Pathogen type:			0.577			0.467
Gram positive	26 (36.6%)	121 (39.2%)		18 (40.9%)	78 (39.4%)	
Gram negative	28 (39.4%)	122 (39.4%)		17 (38.6%)	88 (44.4%)	
Viral	7 (9.8%)	38 (12.3%)		3 (6.8%)	16 (8.1%)	
Fungal	7 (9.8%)	15 (4.8%)		4 (9.0%)	6 (13.6%)	
Mixed	3 (4.2%)	13 (4.2%)		2 (4.5%)	8 (4.1%)	
Comorbidity						
Heart disease	9 (7.1%)	35 (6.7%)	0.869	4 (5.6%)	24 (6.8%)	0.722
Lung disease	12 (9.5%)	50 (9.6%)	0.975	7 (9.8%)	22 (6.2%)	0.281
Neurologic disease	10 (7.9%)	107 (20.6%)	0.001	9 (12.7%)	67 (18.9%)	0.194
Kidney disease	19 (15.1%)	13 (2.5%)	0.001	5 (7.0%)	10 (2.8%)	0.079
Liver disease	10 (7.9%)	25 (4.8%)	0.164	12 (16.9%)	28 (7.9%)	0.018
Solid organ transplant	5 (4.0%)	13 (2.5%)	0.369	4 (5.6%)	16 (4.5%)	0.686
Oncologic disease	26 (20.6%)	56 (10.8%)	0.003	11 (15.5%)	42 (11.9%)	0.398
Bone marrow transplant	17 (13.5%)	22 (4.3%)	<0.001	9 (12.8%)	29 (8.2%)	0.227
PRISM III	16 (9, 24)	11 (6, 16)	<0.001	16 (11, 23)	10 (6, 15)	<0.001
Day 1 VIS	30 (10, 100)	15 (7, 40)	<0.001	40 (13, 150)	16 (8, 31)	<0.001
Day 1 P/F <250	31 (24.6%)	118 (22.7%)	0.648	23 (32.4%)	69 (19.5%)	<0.016
PICU LOS	7 (2, 15)	6 (2, 12)	0.673	7 (2, 14)	5 (2, 11)	0.815
PICU Free days	22 (12, 26)	22 (16, 26)	0.668	23 (32.4%)	23 (17, 26)	0.804
Hospital LOS	14 (5, 28)	13 (7, 27)	0.955	15 (3, 28)	14 (7, 26)	0.441
7-day mortality	31 (24.6%)	27 (5.2%)	<0.001	20 (28.2%)	19 (5.4%)	<0.001
28-day mortality	41 (32.5%)	46 (8.9%)	<0.001	25 (35.2%)	30 (8.5%)	<0.001
Complicated course	75 (59.5%)	138 (26.5%)	<0.001	48 (67.6%)	96 (27.1%)	<0.001
Cardiac arrest	67 (53.2%)	76 (14.6%)	<0.001	38 (53.5%)	55 (15.5%)	<0.001
Day 7 Cardiovascular dysfunction	54 (42.845.5%)	85 (16.4%)	<0.001	36 (50.7%)	71 (20.1%)	<0.001
Day 7 Respiratory Dysfunction	72 (57.2%)	170 (32.7%)	<0.001	46 (64.8%)	120 (33.9%)	<0.001
Day 7 Kidney Dysfunction	64 (50.8%)	104 (20.0%)	<0.001	42 (59.2%)	68 (19.2%)	<0.001
Day 7 Neuro Dysfunction	27 (21.4%)	24 (4.6%)	<0.001	19 (26.8%)	19 (5.4%)	<0.001
Day 7 Hematologic Dysfunction	59 (46.8%)	79 (15.2%)	<0.001	36 (50.7%)	48 (13.6% 0	<0.001
Day 7 Hepatic Dysfunction	50 (39.7%)	57 (11.0%)	<0.001	34 (47.9%)	31 (8.8%)	<0.001
Day 7 Vasoactive support†	28/70 (40.0%)	55/278 (19.7%)	<0.001	15/39 (38.4%)	40/173 (23.1%)	<0.001
Day 7 Mechanical ventilation †	51/70 (72.8%)	164/278 (58.9%)	0.033	30/39 (76.9%)	101/173 (58.3%)	0.031
Day 7 CRRT †	27/70 (38.6%)	22/278 (7.9%)	<0.001	10/39 (25.6%)	12/173 (6.9%)	<0.001
Day 1–7 % positive fluid balance	6.6 (1.9, 16.6%)	4.9 (0.0, 11.7)	0.016	8.3 (1.7, 17.8)	4.9 (0.7, 11.6)	0.008
Any ECMO	2 (1.6%)	1 (0.2%)	0.039	1 (1.4%)	1 (0.3%)	0.345
Corticosteroids	82 (65.1%)	279 (53.7%)	0.020	53 (74.7%)	187 (52.8%)	<0.001

Abbreviations:

PRISM III Pediatric risk of mortality score -III

VIS: Vasoactive inotropic score

P/F: PaO2/FiO2 ratio.

LOS: Length of stay

CRRT: Continuous renal replacement therapy

ECMO: Extracorporeal membrane oxygenation.

**Table 2.** Results of tests for interaction between pediatric septic shock latent profile phenotypes and common sepsis therapies on clinical outcomes.

	Inflamed		Uninflamed		P value
	≥ 100 ml/kg (n=118)	<100 ml/kg (n=77)	≥ 100 ml/kg (n=506)	<100 ml/kg (n=367)	
7-day mortality	35 (29.7%)	14 (18.2%)	25 (4.9%)	20 (5.5%)	0.081
28-day mortality	45 (38.1%)	19 (24.7%)	46 (9.1%)	29 (7.9%)	0.169
Complicated course	84 (71.2%)	37 (48.1%)	161 (31.8%)	72 (19.6%)	0.280
	Inflamed		Uninflamed		P value
	≥ 2ABMs (n=177)	< 2 ABMs (n=20)	≥ 2ABMs (n=793)	< 2 ABMs (n=81)	
7-day mortality	47 (26.6%)	4 (20.0%)	40 (5.1%)	6 (7.4%)	0.316
28-day mortality	61 (34.5%)	5 (25.0%)	67 (8.5%)	9 (11.1%)	0.269
Complicated course	116 (65.5%)	7 (35.0%)	211 (26.6%)	23 (28.4%)	0.021*
	Inflamed		Uninflamed		P value
	≥ 2 VA (n=112)	<2 VA (n=85)	≥ 2 VA (n=344)	<2 VA (n=530)	
7-day mortality	36 (32.2%)	15 (17.7%)	26 (7.6%)	20 (3.8%)	0.908
28-day mortality	44 (39.3%)	22 (25.9%)	37 (10.8%)	39 (7.4%)	0.844
Complicated course	80 (71.4%)	43 (50.6%)	119 (34.6%)	115 (21.7%)	0.622
	Inflamed		Uninflamed		P value
	Steroids (n=135)	No Steroids (n=62)	Steroids (n=466)	No steroids (n=408)	
7-day mortality	40 (29.6%)	11 (17.7%)	34 (7.3%)	12 (2.9%)	0.532
28-day mortality	52 (38.5%)	14 (22.6%)	57 (12.2%)	19 (4.7%)	0.454
Complicated course	95 (70.4%)	28 (45.2%)	147 (31.6%)	87 (21.3%)	0.101
	Inflamed		Uninflamed		P value
	Intubated (n=88)	Not intubated (n=109)	Intubated (n=352)	Not intubated (n=522)	
7-day mortality	31 (35.2%)	20 (18.4%)	28 (7.9%)	18 (3.5%)	0.847
28-day mortality	42 (47.7%)	24 (22.1%)	46 (13.1%)	30 (5.8%)	0.674
Complicated course	71 (80.7%)	52 (47.7%)	136 (38.6%)	98 (18.8%)	0.244
	Inflamed		Uninflamed		P value
	CRRT (n=42)	No CRRT (n=155)	CRRT (n=21)	No CRRT (n=853)	
7-day mortality	15 (35.7%)	36 (23.3%)	1 (4.8%)	45 (5.3%)	0.461
28-day mortality	22 (52.4%)	44 (28.4%)	8 (38.1%)	68 (8.0%)	0.155
Complicated course	34 (80.9%)	89 (57.4%)	14 (66.7%)	220 (25.8%)	0.424

ABM: Anti-microbials.

VA: Vasoactive agents.

Day 1 Intubation.

Day 1 CRRT initiation.

P value for interaction between phenotype and intervention on clinical outcome tested based on logistic regression including age and PRISM III score as covariates

## Data Availability

All de-identified clinical data are available upon reasonable request to the corresponding author. Bulk messenger RNA sequencing (fastq) files and related metadata will be uploaded into GEO upon acceptance of manuscript for publication. The R code for methods detailed in the manuscript are available at ***

## Research in context

### Evidence before this study:

Clinical and biological heterogeneity among critically ill patients has impeded the discovery of efficacious therapeutic approaches against sepsis. Thus, reproducible strategies to identify biologically relevant subclasses of patients are the need of the hour.

### Added value of this study:

Through latent profile analyses, we identified two reproducible phenotypes of pediatric septic shock –’inflamed’ and ‘uninflamed’ – of high prognostic value. The ‘inflamed’ phenotype was characterized primarily by activation of the innate immune system. The newly derived phenotypes did not show significant overlap with established gene-expression-based pediatric septic shock endotypes, which are reflective of the adaptive immune system. An integrated subclassification scheme that considered both endotype and phenotype demonstrated varying survival probabilities and holds potential therapeutic relevance.

### Implications of all available evidence:

Integrated approaches to simultaneously sample various biological facets of host response in sepsis hold the potential to offer a deeper understanding of human sepsis heterogeneity. Pending validation, such a comprehensive understanding of patient-level pathobiology among septic children may lead to the targeted delivery of sepsis therapies.

## References

[1] Rudd KE, Johnson SC, Agesa KM, Shackelford KA, Tsoi D, Kievlan DR, Global, regional, and national sepsis incidence and mortality, 1990–2017: analysis for the Global Burden of Disease Study. The Lancet. 2020 Jan;395(10219):200–11. 10.1016/S0140-6736(19)32989-7 PMC6970225 31954465

[2] Global report on the epidemiology and burden of sepsis: current evidence, identifying gaps and future directions. World Health Organization. 2020. [Internet]. p. p.56. Available from: https://apps.who.int/iris/bitstream/handle/10665/334216/9789240010789-eng.pdf?sequence=1&isAllowed=y

[3] Marshall JC. Why have clinical trials in sepsis failed? Trends Mol Med. 2014 Apr;20(4):195–203. 24581450 10.1016/j.molmed.2014.01.007

[4] Shah FA, Meyer NJ, Angus DC, Awdish R, Azoulay É, Calfee CS, A Research Agenda for Precision Medicine in Sepsis and Acute Respiratory Distress Syndrome: An Official American Thoracic Society Research Statement. Am J Respir Crit Care Med. 2021 Oct 15;204(8):891–901. 34652268 10.1164/rccm.202108-1908ST PMC8534611

[5] Wong HR, Cvijanovich N, Lin R, Allen GL, Thomas NJ, Willson DF, Identification of pediatric septic shock subclasses based on genome-wide expression profiling. BMC Med. 2009 Jul 22;7:34. 19624809 10.1186/1741-7015-7-34 PMC2720987

[6] Davenport EE, Burnham KL, Radhakrishnan J, Humburg P, Hutton P, Mills TC, Genomic landscape of the individual host response and outcomes in sepsis: a prospective cohort study. Lancet Respir Med. 2016 Apr;4(4):259–71. 26917434 10.1016/S2213-2600(16)00046-1 PMC4820667

[7] Scicluna BP, van Vught LA, Zwinderman AH, Wiewel MA, Davenport EE, Burnham KL, Classification of patients with sepsis according to blood genomic endotype: a prospective cohort study. Lancet Respir Med. 2017 Oct;5(10):816–26. 28864056 10.1016/S2213-2600(17)30294-1

[8] Sweeney TE, Perumal TM, Henao R, Nichols M, Howrylak JA, Choi AM, A community approach to mortality prediction in sepsis via gene expression analysis. Nat Commun. 2018 Feb 15;9(1):694. 29449546 10.1038/s41467-018-03078-2 PMC5814463

[9] Wong HR, Cvijanovich N, Lin R, Allen GL, Thomas NJ, Willson DF, Identification of pediatric septic shock subclasses based on genome-wide expression profiling. BMC Medicine. 2009 Jul 22;7(1):34. 19624809 10.1186/1741-7015-7-34 PMC2720987

[10] Wong HR, Cvijanovich NZ, Anas N, Allen GL, Thomas NJ, Bigham MT, Developing a clinically feasible personalized medicine approach to pediatric septic shock. Am J Respir Crit Care Med. 2015 Feb 1;191(3):309–15. 25489881 10.1164/rccm.201410-1864OC PMC4351580

[11] Antcliffe DB, Burnham KL, Al-Beidh F, Santhakumaran S, Brett SJ, Hinds CJ, Transcriptomic Signatures in Sepsis and a Differential Response to Steroids. From the VANISH Randomized Trial. Am J Respir Crit Care Med. 2019 Apr 15;199(8):980–6. 30365341 10.1164/rccm.201807-1419OC PMC6467319

[12] Calfee CS, Delucchi K, Parsons PE, Thompson BT, Ware LB, Matthay MA, Subphenotypes in acute respiratory distress syndrome: latent class analysis of data from two randomised controlled trials. Lancet Respir Med. 2014 Aug;2(8):611–20. 24853585 10.1016/S2213-2600(14)70097-9 PMC4154544

[13] Calfee CS, Delucchi KL, Sinha P, Matthay MA, Hackett J, Shankar-Hari M, ARDS Subphenotypes and Differential Response to Simvastatin: Secondary Analysis of a Randomized Controlled Trial. Lancet Respir Med. 2018 Sep;6(9):691–8. 30078618 10.1016/S2213-2600(18)30177-2 PMC6201750

[14] Sinha P, Furfaro D, Cummings MJ, Abrams D, Delucchi K, Maddali MV, Latent Class Analysis Reveals COVID-19-related Acute Respiratory Distress Syndrome Subgroups with Differential Responses to Corticosteroids. Am J Respir Crit Care Med. 2021 Dec 1;204(11):1274–85. 34543591 10.1164/rccm.202105-1302OC PMC8786071

[15] Dahmer MK, Yang G, Zhang M, Quasney MW, Sapru A, Weeks HM, Identification of phenotypes in paediatric patients with acute respiratory distress syndrome: a latent class analysis. Lancet Respir Med. 2022 Mar;10(3):289–97. 34883088 10.1016/S2213-2600(21)00382-9 PMC8897230

[16] Yehya N, Zinter MS, Thompson JM, Lim MJ, Hanudel MR, Alkhouli MF, Identification of molecular subphenotypes in two cohorts of paediatric ARDS. Thorax. 2023 Oct 9;thorax-2023-220130. 10.1136/thorax-2023-220130 PMC10850835 37813544

[17] Sinha P, Kerchberger VE, Willmore A, Chambers J, Zhuo H, Abbott J, Identifying molecular phenotypes in sepsis: an analysis of two prospective observational cohorts and secondary analysis of two randomised controlled trials. The Lancet Respiratory Medicine [Internet]. 2023 Aug 23 [cited 2023 Aug 25];0(0). Available from: https://www.thelancet.com/journals/lanres/article/PIIS2213-2600(23)00237-0/fulltext 10.1016/S2213-2600(23)00237-0 PMC10841178 37633303

[18] Wong HR, Caldwell JT, Cvijanovich NZ, Weiss SL, Fitzgerald JC, Bigham MT, Prospective clinical testing and experimental validation of the Pediatric Sepsis Biomarker Risk Model. Sci Transl Med [Internet]. 2019 Nov 13 [cited 2021 Mar 15];11(518). Available from: https://www.ncbi.nlm.nih.gov/pmc/articles/PMC7720682/10.1126/scitranslmed.aax9000 PMC7720682 31723040

[19] Atreya MR, Cvijanovich NZ, Fitzgerald JC, Weiss SL, Bigham MT, Jain PN, Integrated PERSEVERE and endothelial biomarker risk model predicts death and persistent MODS in pediatric septic shock: a secondary analysis of a prospective observational study. Critical Care. 2022 Jul 11;26(1):210. 35818064 10.1186/s13054-022-04070-5 PMC9275255

[20] Goldstein B, Giroir B, Randolph A, International Consensus Conference on Pediatric Sepsis. International pediatric sepsis consensus conference: definitions for sepsis and organ dysfunction in pediatrics. Pediatr Crit Care Med. 2005 Jan;6(1):2–8. 15636651 10.1097/01.PCC.0000149131.72248.E6

[21] Schulte-Schrepping J, Reusch N, Paclik D, Baßler K, Schlickeiser S, Zhang B, Severe COVID-19 Is Marked by a Dysregulated Myeloid Cell Compartment. Cell. 2020 Sep 17;182(6):1419–1440.e23. 32810438 10.1016/j.cell.2020.08.001 PMC7405822

[22] Wilk AJ, Lee MJ, Wei B, Parks B, Pi R, Martínez-Colón GJ, Multi-omic profiling reveals widespread dysregulation of innate immunity and hematopoiesis in COVID-19. J Exp Med. 2021 Aug 2;218(8):e20210582. 34128959 10.1084/jem.20210582 PMC8210586

[23] Sinha S, Rosin NL, Arora R, Labit E, Jaffer A, Cao L, Dexamethasone modulates immature neutrophils and interferon programming in severe COVID-19. Nat Med. 2022 Jan;28(1):201–11. 34782790 10.1038/s41591-021-01576-3 PMC8799469

[24] Pollack MM, Patel KM, Ruttimann UE. The Pediatric Risk of Mortality III--Acute Physiology Score (PRISM III-APS): a method of assessing physiologic instability for pediatric intensive care unit patients. J Pediatr. 1997 Oct;131(4):575–81. 9386662 10.1016/s0022-3476(97)70065-9

[25] Heijnen NFL, Hagens LA, Smit MR, Cremer OL, Ong DSY, van der Poll T, Biological Subphenotypes of Acute Respiratory Distress Syndrome Show Prognostic Enrichment in Mechanically Ventilated Patients without Acute Respiratory Distress Syndrome. Am J Respir Crit Care Med. 2021 Jun 15;203(12):1503–11. 33465019 10.1164/rccm.202006-2522OC

[26] Bhatraju PK, Zelnick LR, Herting J, Katz R, Mikacenic C, Kosamo S, Identification of Acute Kidney Injury Subphenotypes with Differing Molecular Signatures and Responses to Vasopressin Therapy. Am J Respir Crit Care Med. 2019 Apr 1;199(7):863–72. 30334632 10.1164/rccm.201807-1346OC PMC6444649

[27] Bos LDJ, Scicluna BP, Ong DSY, Cremer O, van der Poll T, Schultz MJ. Understanding Heterogeneity in Biologic Phenotypes of Acute Respiratory Distress Syndrome by Leukocyte Expression Profiles. Am J Respir Crit Care Med. 2019 Jul;200(1):42–50. 30645145 10.1164/rccm.201809-1808OC

[28] Kwok AJ, Allcock A, Ferreira RC, Cano-Gamez E, Smee M, Burnham KL, Neutrophils and emergency granulopoiesis drive immune suppression and an extreme response endotype during sepsis. Nat Immunol. 2023 May;24(5):767–79. 37095375 10.1038/s41590-023-01490-5

[29] Grooth HJ de Cremer OL. Beyond patterns: how to assign biological meaning to ARDS and sepsis phenotypes. The Lancet Respiratory Medicine [Internet]. 2023 Aug 23 [cited 2023 Oct 24];0(0). Available from: https://www.thelancet.com/journals/lanres/article/PIIS2213-2600(23)00266-7/fulltext 10.1016/S2213-2600(23)00266-7 37633305

[30] van Amstel RBE, Kennedy JN, Scicluna BP, Bos LDJ, Peters-Sengers H, Butler JM, Uncovering heterogeneity in sepsis: a comparative analysis of subphenotypes. Intensive Care Med [Internet]. 2023 Oct 18 [cited 2023 Oct 23]; Available from: 10.1007/s00134-023-07239-w PMC10622359 37851064

